# Poster Session II - A295 IMPACT OF INFLAMMATORY BOWEL DISEASE ON ANXIETY AND DEPRESSION DURING PREGNANCY: A RETROSPECTIVE SURVEY STUDY

**DOI:** 10.1093/jcag/gwaf042.295

**Published:** 2026-02-13

**Authors:** N Butani, S Perera, K O’ Connor, K Xiao, V Srikanth, V Premjeyanth, Y Patel, C Maxwell, N Griller, T Zenlea, V W Huang

**Affiliations:** University of Toronto, Toronto, ON, Canada; University of Toronto, Toronto, ON, Canada; University of Toronto, Toronto, ON, Canada; University of Toronto, Toronto, ON, Canada; University of Toronto, Toronto, ON, Canada; University of Toronto, Toronto, ON, Canada; University of Toronto, Toronto, ON, Canada; University of Toronto, Toronto, ON, Canada; University of Toronto, Toronto, ON, Canada; University of Toronto, Toronto, ON, Canada; University of Toronto, Toronto, ON, Canada

## Abstract

**Background:**

Individuals with inflammatory bowel disease (IBD) experience mental health disorders at a rate of 1.5 to 2 times higher than the general population. With the rising incidence of IBD, many people are affected by the disease during significant life stages such as pregnancy. Research on the impact of IBD on anxiety and depression in pregnant people is limited.

**Aims:**

To assess the impact of active IBD on anxiety and depression during pregnancy.

**Methods:**

This retrospective survey study analyzed data from patients seen at the ‘Preconception and Pregnancy in IBD’ clinic at Mount Sinai Hospital who conceived prior to January 1^st^, 2022. Standardized pre-visit surveys included validated measures of mental health (General Anxiety Disorder-7 for anxiety; Patient Health Questionnaire-9 for depression) and IBD disease activity (pMayo6 for ulcerative colitis (UC); mHBI for Crohn’s disease (CD)). Demographic data was also collected (age, marital status, occupational status, smoking habits, alcohol use, cannabis use, previous diagnosis of anxiety and/or depression, IBD type, IBD surgical history, and medication use). Linear regression models were used to assess associations between disease activity and anxiety/depression, adjusting for medication type and stratified by trimester and IBD subtype.

**Results:**

A total of 94 patients were included in this study, 42 patients had UC (44.7%), and 52 patients had CD (55.3%). The median age for the UC group was 32 (IQR 31-35.8), and the median age for the CD group was 32.7 (IQR 30-35.2).

Active CD in the second trimester was significantly associated with higher anxiety (p = 0.024) and depression scores (p = 0.021) compared to those in remission. For CD, 6 patients (35.3%) in trimester 1, 8 patients in trimester 2 (25.8%), and 8 patients (26.7%) in trimester 3 had a GAD-7 score of ≥ 5; 5 patients (29.4%) in trimester 1, 6 patients (19.4%) in trimester 2, and 7 patients (23.3%) in trimester 3 had a PHQ-9 score of ≥ 5.

Active UC in the second trimester was associated with significantly higher anxiety (p = 0.034) and depression (p = 0.022) scores compared to inactive UC. In the third trimester, this pattern persisted, with active UC again associated with elevated anxiety (p = 0.030) and depression (p = 0.027) scores. For UC, 4 patients (33.3%) in trimester 1, 9 patients (30%) in trimester 2, and 9 patients (31.0%) in trimester 3 had a GAD-7 score of ≥ 5; 4 patients in trimester 1, 4 patients in trimester 2, and 7 patients in trimester 3 had a PHQ-9 score of ≥ 5.

**Conclusions:**

Active IBD during pregnancy is significantly associated with increased anxiety and depression, therefore, a multidisciplinary approach including mental health support is imperative for pregnant people with IBD.

**Funding Agencies:**

None

